# 4259 Application of Design Sprint Methodology to Prototype a Proactive Outreach Tool for COPD Patients

**DOI:** 10.1017/cts.2020.55

**Published:** 2020-07-29

**Authors:** Michael Cui, Lindsay Zimmerman, Shashin Chokshi

**Affiliations:** 1 University of Chicago

## Abstract

OBJECTIVES/GOALS: The primary objective of this study was to apply design sprint methodology to develop a proactive outreach tool prototype for patients with chronic obstructive pulmonary disease (COPD). METHODS/STUDY POPULATION: We utilized a 3-day process to align our team and key stakeholders behind answering the following question: “how might we empower COPD patients to understand their healthcare information, make decisions in partnership with their providers, and more easily manage their daily health?” On Day 1, we focused on understanding and defining the problem, and mapping the patient experience. On Day 2, we quickly brainstormed potential solutions, sketched our top ideas, and listed the solutions’ inherent assumptions. On Day 3, we created a prototype of our top solution and storyboarded each step of the prototype experience to review its potential usability and comprehensibility with patients. RESULTS/ANTICIPATED RESULTS: At the end of the design sprint, our team developed a prototype centered around personalized communication between COPD patients and providers. The prototype focuses on augmenting the current transitional care management (TCM) workflow in the post-discharge period. We are working to further develop the prototype prior to formal testing with care coordinators and patients. We anticipate that our prototype will assist in automating the current TCM workflow and facilitate contact with more patients post-discharge. DISCUSSION/SIGNIFICANCE OF IMPACT: Contact with patients is currently challenging due limited resources and the time sensitive nature of the TCM requirements. Automated patient outreach may be especially effective in engaging patients on a large scale, while also minimizing time and resources needed from healthcare staff.

